# Retraction Note: Jacarelhyperol A induced apoptosis in leukaemia cancer cell through inhibition the activity of Bcl-2 proteins

**DOI:** 10.1186/s12885-022-09868-8

**Published:** 2022-07-12

**Authors:** Shoude Zhang, Jun Yin, Xia Li, Jigang Zhang, Rongcai Yue, Yanyan Diao, Honglin Li, Hui Wang, Lei Shan, Weidong Zhang

**Affiliations:** 1 School of Pharmacy, Second Military Medical University, 325# Guohe Road, Shanghai, 200433 China; 2 Shanghai Key Laboratory of New Drug Design, State Key Laboratory of Bioreactor Engineering, School of Pharmacy, East China University of Science and Technology, 130# Meilong Road, Shanghai, 200237 China; 3 Key Laboratory of Nutrition and Metabolism, Institute for Nutritional Sciences, Shanghai Institutes for Biological Sciences, Chinese Academy of Sciences, Graduate School of the Chinese Academy of Sciences, 320# YueYang Road, Shanghai, 201203 China

**Retraction Note: BMC Cancer 14, 689 (2014)**

**https://doi.org/10.1186/1471-2407-14-689**

The Editors have retracted this article. After publication, concerns were raised over what appears to be repeated elements between plots in Figure 3 and unexpected overlaps in Figs. 4A and 5A and B. The authors have been unable to supply the original data for the article and requested a retraction. Therefore, the Editors have lost confidence in the integrity of the results.

Shoude Zhang, Xia Li, Jigang Zhang, Rongcai Yue, Yanyan Diao, Honglin Li, Lei Shan and Weidong Zhang agree to this retraction. Jun Yin and Hui Wang have not responded to any correspondence from the editor about this retraction.

